# PHASTpep: Analysis Software for Discovery of Cell-Selective Peptides via Phage Display and Next-Generation Sequencing

**DOI:** 10.1371/journal.pone.0155244

**Published:** 2016-05-17

**Authors:** Lindsey T. Brinton, Dustin K. Bauknight, Siva Sai Krishna Dasa, Kimberly A. Kelly

**Affiliations:** 1 Department of Biomedical Engineering, University of Virginia, Charlottesville, Virginia, 22908, United States of America; 2 Cardiovascular Research Center, University of Virginia, Charlottesville, Virginia, 22908, United States of America; Northwestern University Feinberg School of Medicine, UNITED STATES

## Abstract

Next-generation sequencing has enhanced the phage display process, allowing for the quantification of millions of sequences resulting from the biopanning process. In response, many valuable analysis programs focused on specificity and finding targeted motifs or consensus sequences were developed. For targeted drug delivery and molecular imaging, it is also necessary to find peptides that are selective—targeting only the cell type or tissue of interest. We present a new analysis strategy and accompanying software, PHage Analysis for Selective Targeted PEPtides (PHASTpep), which identifies highly specific and selective peptides. Using this process, we discovered and validated, both in vitro and in vivo in mice, two sequences (HTTIPKV and APPIMSV) targeted to pancreatic cancer-associated fibroblasts that escaped identification using previously existing software. Our selectivity analysis makes it possible to discover peptides that target a specific cell type and avoid other cell types, enhancing clinical translatability by circumventing complications with systemic use.

## Introduction

Phage display enables the simultaneous, high throughput screening of billions of different peptides against diverse target molecules including proteins [1], miRNAs [2], polysaccharides [3], cells [4], and tissues [5]. Phage display’s utility and versatility have made it an important technique in the identification of molecularly targeted affinity agents for imaging, targeted drug therapy, and biosensor applications [6]. In addition to the biomedical field, phage display derived peptides that bind certain types of inorganic materials have been identified, such as gold-binding sequences, and have established insight into ligand recognition mechanisms and quantitative affinity analysis [7]. Further, phage display has been used to develop self-assembling batteries [8].

Phage display offers a number of important advantages in identifying targeted peptides such as rapid and economical biological expansion, vast peptide diversity, a rapid screening process, and the availability of many types of phage clones and libraries (for review see [6]). Another important advantage is that bacteriophage, unlike higher organisms, have only one copy of each gene, so it is easy to identify the displayed peptide of a clone by sequencing the appropriate portion of the phage genome. In a process called biopanning, a phage library is exposed to a target, non-bound phage are washed away, and bound phage are eluted off then amplified in their bacterial host. Theoretically, the amplified library has an increased proportion of phage clones that bind the target and can be further enriched by being subjected to additional rounds of panning. However, traditional screening protocols are hampered by false positive rates caused by non-specific phage binding and unequal rates of amplification [9] as well as by loss of potential candidates early in the process due to low starting phage concentrations. Following the iterative selection process, it is necessary to differentiate between phage clones that bind the target specifically and those that have been co-extracted in the enriched library due to non-specific interactions. Initially, the standard technique was to pick and amplify individual clones from the final enriched phage pool and test them side-by-side in an ELISA to distinguish the specific binders. The identity of the displayed peptides with specific binding could be individually determined by Sanger sequencing.

Software programs have been developed to assist in the analysis of peptides identified through phage display. One of the earliest programs, REceptor LIgand Contacts (RELIC) [10] is still popular in the phage display community. Although RELIC was limited by biases associated with the original phage display technique, it made the great contribution of enabling users to align sequences and find motifs from phage display experimental results. Since RELIC, databases such as PEPBANK [11] and MimoDB [12], have been generated to browse for similar peptide sequences among those compiled from previously-conducted biopanning experiments. Other algorithms and programs were used to analyse results via methods that aligned sequences, performed epitope-mapping, and identified motifs, including MIMOP [13], PEPTIDE [14], SiteLight [15], Multiple Em for Motif Elicitation (MEME) [16], DNAStar [17], Short Linear Motif Finder (SLiMFinder) [18], and Multiple Alignment using Fast Fourier Transform (MAFFT) [19]. Importantly, all of these methods were designed to interpret the small-scale results of traditional phage display. Thus they were still susceptible to many of the aforementioned weaknesses of the technique and a much deeper characterization of the post-biopanning enriched phage library would later prove to further enhance analytic capabilities.

The necessary deeper characterization became attainable with the advent of next generation sequencing (NGS) techniques, whereby it became possible to increase the yield of sequences from hundreds to millions and overcome some of the drawbacks of phage display. NGS has similarly been employed in other *in vitro* selection fields, including yeast display [20], mRNA display [21], antibody display [22–23], protein domains [24], and aptamer selection [25–27]. In the peptide phage display field, NGS has highlighted the differences in amplification rates among phage clones that culminate in domination of the final enriched phage libraries by so-called parasitic sequences [9], leading to development of techniques that use modified amplification techniques or only one round of panning in order to circumvent this amplification bias [21, 28–29]. The effect of sequencing errors in the context of phage display has also been explored [30]. Most importantly, the use of NGS with phage has been successful in moving forward the field of phage display [31–33]. Yet, NGS also introduced the challenge of determining which sequences represent the most ideal ligands since the results still conflate enrichment due to ligand specificity with the enrichment due to non-specific binding and, when more than one round of panning is necessary, variation in amplification rate.

Since the onset of deep sequencing techniques, exquisite work has been carried out to build software for processing and translation of sequences and finding consensus sequences or motifs, including MATLAB-based translation software [34], Multiple Specificity Identifier (MUSI) [35], and target-binding motif analysis [29]. Additionally, powerful analytic methods and software have been developed for processing data, clustering sequences and comparing selective versus non-selective libraries [36–37], affinity ranking [38], and statistical-based comparisons [21]. While these tools provide valuable insight into the specificity and affinity of peptides, they do not address selectivity. Selectivity is an especially important parameter for use of phage display in finding clinically relevant targeted peptides. For example, phage display has been used to identify targeted peptides that can distinguish between diseased and healthy cells [39–41], which in turn can be used for molecular imaging or to alter the toxicity profile of drugs via targeted delivery. Due to the physiologic presence of numerous cell types and receptors, it is necessary to ensure that a given peptide is not only specific for the target of interest but also that it will *not* bind to other locations (i.e., the molecular target is only present on the cell of interest).

Here we present our efficient methodology and accompanying software that rapidly generates highly specific and selective peptides. Our approach can be universally applied from simple purified protein screens to more complex in vitro cell, in vivo, or ex vivo tissue screening and does not require a priori knowledge of a target receptor. Our system consists of (1) independent positive and negative screens, (2) data normalization to total read number and amplified, unselected library, (3) sorting by the ratio of average normalized frequency for positive screens to negative screens, and (4) selection of peptides with highest selectivity (high normalized frequencies in at least two of three positive screens and low normalized frequencies in all negative screens). Although one round of panning has proved successful in other contexts, we determined that the enrichment accomplished by an additional round of panning aided in identification of peptides, especially when selecting on cells. We found that normalizing the data to an amplified unselected library successfully avoids nonspecific rapid amplifiers, or parasitic phage. The software that we present, PHage Analysis for Selective Targeted PEPtides (PHASTpep), is MATLAB-based with a graphical user interface that guides users through two parts of analysis. The first part of our software allows the importation of sequences directly from the fastq file, pulls out the portion of DNA corresponding to the displayed peptides, translates the sequences into amino acids, and calculates the frequency of each unique peptide. The second part of our software normalizes each screen to its read depth and an amplified, unselected library (named reference library), then generates a matrix where each row is a unique peptide sequence and each column is a positive or negative screen. The matrix is sorted according to the average normalized frequency across positive screens divided by the average normalized frequency across negative screens in order to distil the ideal peptide candidates to the top.

We demonstrate our technique in the typified streptavidin protein setting and in the clinically relevant identification of peptides selective for cancer-associated fibroblasts (CAFs) isolated from the stroma of patients with pancreatic adenocarcinoma (PDAC); subsequently validating CAF targeting both in vitro and in vivo in mice. While available motif or consensus-based software excels at narrowing down specificity and identifies the HPQ-motif from a Streptavidin screen, such software found few or no valid motifs in the CAF data, potentially because of the more complex cell environment. Our selectivity analysis is able to distil out the sequences selective for one cell type over several others and identify candidate sequences that we were able to validate in vitro and in vivo. Our CAF-targeted peptides demonstrate the ability of our approach to identify selective peptides despite the challenge of differentiating between similar cells types like normal fibroblasts from which CAFs derive or other pancreatic-specific cells. In application to PDAC, our targeted peptides are a new tool for tackling the devastating disease with a 5-year survival rate of merely 6% and a lack of improvement spanning the past few decades [42].

## Results

### Biopanning and NGS analysis

The commercially available PhD7 library was panned against a positive target as well as negative targets that were used to eliminate non-selective peptide sequences. We carried out two sets of screens. One set was aimed at Streptavidin protein as a positive target, with Vascular Cell Adhesion Molecule (VCAM) protein serving as a negative target (Table 1). Another screen used CAFs as the positive target, with a variety of negative target cells: normal fibroblasts (MRC5), normal pancreatic ductal epithelial cells (HPDE), normal endothelial cells (HUVEC), tumor-conditioned endothelial cells (HUVEC-TCM), and three types of pancreatic tumor epithelial cells (BXPC3, YAPC, L3.6 PL) (Table 2). Phage were eluted with a non-specific low pH glycine wash with the exception of two of the streptavidin screens where we added an additional level of comparison by displacing phage specifically via a biotin wash (per NEB’s guidelines). For each screen, two rounds of biopanning were completed, then phage DNA was isolated after bacterial incubation, PCR amplified, and analysed in an Illumina MiSeq sequencer. Selectivity analysis across positive and negative screens was carried out using the second round data. The Illumina sequencer sorts sequences by barcode into fastq files, which we used as the input for our software.

**Table 1:** Summary of Streptavidin screens. Positive and negative screens were carried out for a protein target, Streptavidin, and processed with PHASTpep.

Screen	Target description	Total reads	Unique reads	Run time (s)
(+) Streptavidin 1	Streptavidin protein	1480544	133633	287
(+) Streptavidin 2	Streptavidin protein	1428274	161291	276
(+) Streptavidin 3	Streptavidin protein	1666760	176704	322
(+) Streptavidin 4	Streptavidin protein	1760774	207670	340
(-) VCAM 1	Vascular Cell Adhesion Molecule	787976	162489	154
(-) VCAM 2	Vascular Cell Adhesion Molecule	951133	211741	185
(-) VCAM 3	Vascular Cell Adhesion Molecule	957800	179189	187

**Table 2:** Summary of CAF screens. Positive and negative screens were carried out for a cell target, CAFs, and processed with PHASTpep.

Screen	Target description	Total reads	Unique reads	Run time (s)
(+) CAF 1	Human Cancer-Associated Fibroblast cells from PDAC patients	2106522	628893	400
(+) CAF 2	Human Cancer-Associated Fibroblast cells from PDAC patients	2194679	495842	414
(+) CAF 3	Human Cancer-Associated Fibroblast cells from PDAC patients	2371639	591354	451
(-) MRC5	Human embryonic lung fibroblast cells	2439664	679702	466
(-) HPDE	Human Pancreatic Ductal Epithelial cells	3098690	889169	653
(-) HUVEC	Human Umbilical Vein Endothelial Cells	823013	113366	149
(-) HUVEC-TCM	Human Umbilical Vein Endothelial Cells in Tumor Conditioned Media	875145	138044	159
(-) BXPC3	Human pancreatic tumor epithelial cells	1032584	148313	200
(-) YAPC	Human pancreatic tumor epithelial cells	919508	145464	172
(-) L3.6 PL	Human pancreatic tumor epithelial cells	1238625	201710	231

**Fig 1:**
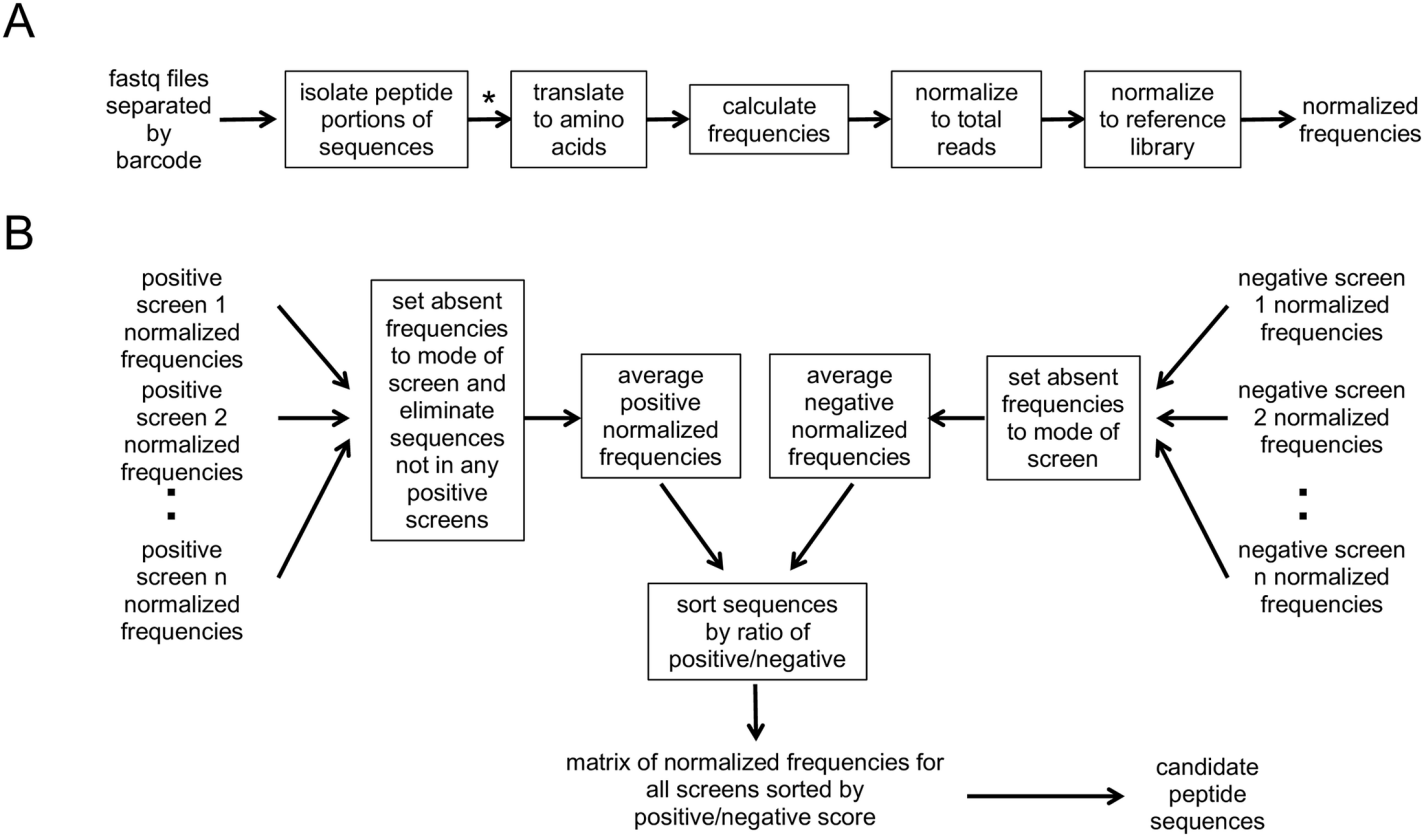
Approach to finding candidate peptide sequences. (A) The Illumina sequencer outputs fastq files that are separated by barcodes. For each of these files, the portion of DNA corresponding to the displayed peptides was isolated and translated. The number of times each sequence was read in a run was summed to obtain the frequency associated with that sequence, which was subsequently divided by the total number of reads from the run and then by the frequency of that sequence in the reference library. This processing resulted in a normalized frequency for each sequence of a run. (B) Sequences present in one screen but absent in another were set to the non-zero mode of the absent screen rather than zero to prevent later division by zero. The normalized frequencies across all positive screens were averaged as well as across all negative screens. The average positive normalized frequency was divided by the average negative normalized frequency and this ratio was used to sort the sequences so that sequences high across positive screens and low across negative screens distilled to the top fraction. Sequences ordered by ratio created the rows of the comparison matrix showing all of the normalized frequencies for each sequence across all screens, facilitating identification of the most selective sequences. * PhD libraries from NEB are generated with constrained codons. When using this library, sequences containing codons not represented in the library are removed.

**Fig 2:**
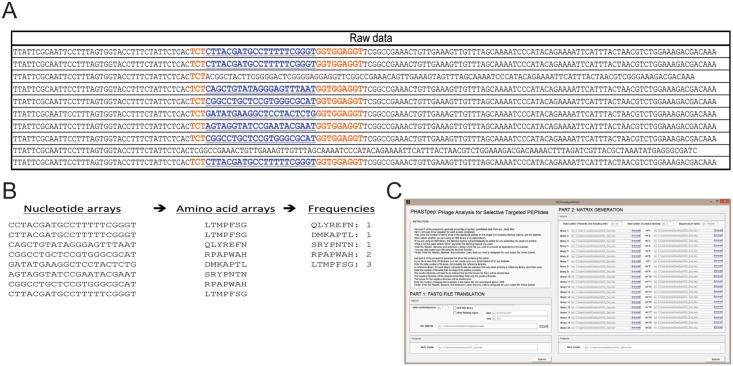
Validation of PHASTpep software translation and frequency calculations. (A) The raw data pulled from the fastq file of the Illumina sequencer showing the unique flanking regions (red) surrounding the portion of the DNA sequence corresponding to the displayed peptides (blue). (B) The unique flanking regions were used to isolate the peptide sequences, which were then translated into amino acids. For each sequence, a frequency was calculated corresponding to the number of times it appeared in the run. (C) GUI of the PHASTpep software presented in this paper to automate the data processing and analysis.

Our analysis process generates normalized frequencies for each fastq file (individual screen) by isolating the portion of the DNA sequence corresponding to the displayed peptides, optionally removing sequences that do not correspond to constrained codons present in NEB libraries, translating it to amino acids, calculating the frequencies, and normalizing to the total number of reads as well as reference library frequencies for peptides (described in detail in the next section) (Fig 1A). The data is then sorted so that sequences selective for a target rise to the top fraction. Normalized frequencies from positive screens are averaged and likewise for negative screens. In the event that a sequence is present in the one screen and absent in another, the absent frequency is replaced by the mode of that screen. The sequences are then sorted by the ratio of the average positive to negative normalized frequency (Fig 1B). Other MATLAB files available from the Heinis lab can input non-segregated Ion Torrent fastq files and generate files sorted by barcode [29], which can subsequently be combined with our selectivity analysis (PHASTpep part two). Because we used the NEB libraries, raw data from the Illumina sequencer contained 21-nucleotide regions corresponding to the displayed peptide flanked by unique nucleotide regions ‘TCT’ and ‘GGAGGTGGA’ (Fig 2A). Part one of PHASTpep successfully isolated and translated the portion of DNA corresponding to the displayed peptides and then calculated the frequency of each sequence, or number of times it was read by the sequencer (Fig 2B). Our software is user friendly, with a graphical user interface designed to guide users through the analysis process and facilitate the use of MATLAB for non-experts (Fig 2C).

### Frequency normalization enables sorting based on interscreen comparisons and identifies the best candidate peptide sequences

**Fig 3:**
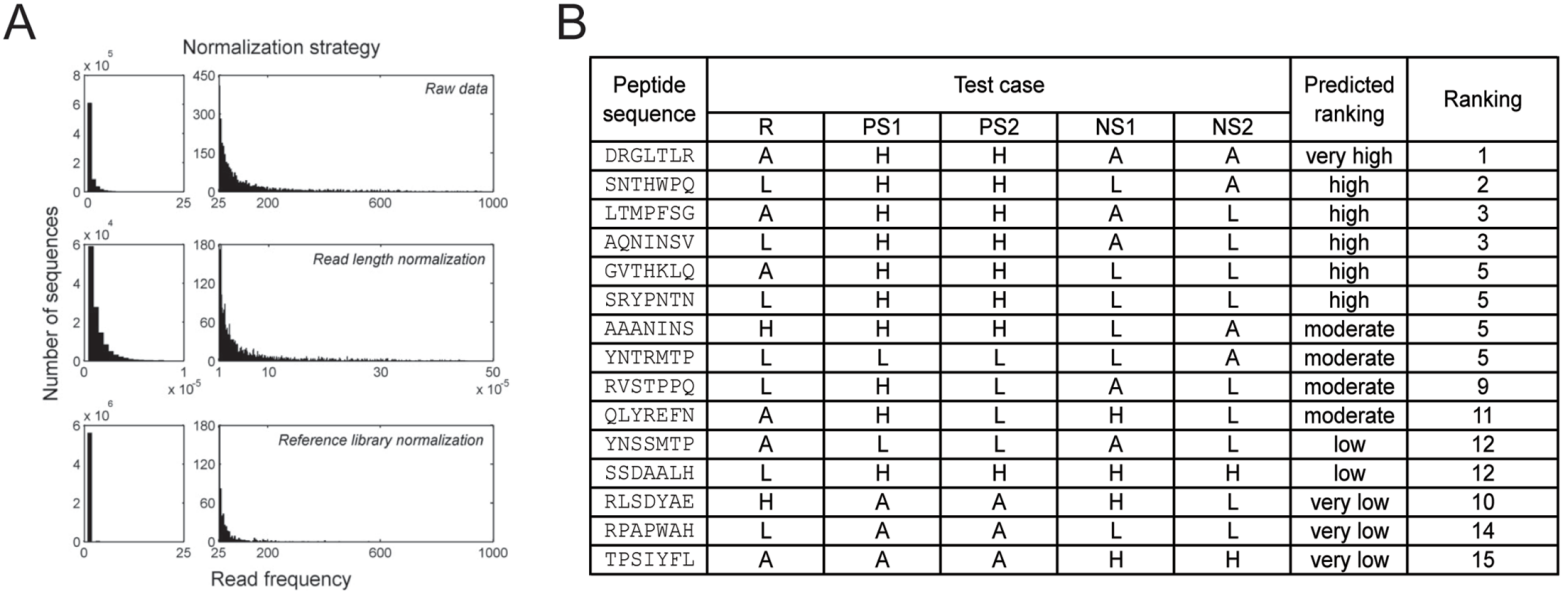
Normalization strategy and sorting of PHASTpep. (A) For each screen, the frequencies were divided by the total number of reads of the screen, followed by the frequency of that sequence in the reference library. (B) In order to demonstrate the sorting process, small libraries were created that represented a reference library, two positive screens, and 2 negative screens. For each sequence, a qualitative ranking was determined (predicted ranking) based on the level of frequency assigned in each library. For example, GVTHKLQ was absent in the reference library, high in both positive screens, and absent in both negative screens. Therefore, it was predicted to be ranked very high. Conversely, TPSIYFL was only high in the negative screens and absent elsewhere. Thus it was predicted to rank very low. For each test case (sequence), the predicted ranking was compared to the actual ranking after running the test libraries through our sorting software. R, reference; PS, positive screen; NS, negative screen; A, absent; L, low; H, high.

To compare peptide frequencies between multiple independent screens, we accounted for two variables: (1) differences in the total number of reads, and (2) differences in underlying phage distributions. Even when using approximately equimolar amounts of DNA, the total number of reads for each barcode may vary. Additionally, from one run to the next, different numbers of lanes may be included, resulting in further discrepancy in the total number of reads between different screens. Thus, the first step of our normalization process is to divide each frequency by the total number of reads from that screen, which acts as a scaling factor (Fig 3A). Secondly, the starting distribution of phage within the NEB libraries has been shown to be unequal [9]. The distribution is further skewed from normal by biases related to bacterial amplification. While some groups have been able to eliminate this problem by carrying out only one round of biopanning [21, 28–29], we found that the enrichment provided by an additional round of biopanning was valuable when working with cells. We hypothesize that while the selectivity of the library is increased by one round of biopanning, the specific binders are distributed among many groups corresponding to the many targets available on a cell, making overrepresentation in the results less evident. Therefore, we elected to do two rounds of biopanning and to lessen the impact of the non-normal underlying distribution and amplification biases with a second normalization step. In this step, an aliquot of unselected library was amplified and sequenced along with the screens, providing a “reference library” with background values for sequences that are overrepresented in the library for reasons other than affinity selection. Frequencies for each sequence were thus divided by the total number of reads and by the corresponding frequency (also normalized to total read number) from the reference library (Fig 3A). It is this set of normalized frequencies that was then used to calculate average positive to negative ratios and sort the data.

We tested the sorting capability of our software using a series of test cases for which is it simple to visually inspect the numbers and determine how ideal the sequences are. In order to simulate the screening and sorting process in a simplified setting, each sequence was assigned a frequency of absent (0, A), low (10, L), or high (1000, H) in a reference library, two positive screens, and two negative screens (Fig 3B). Thus applying predictive logic to the patterns of the test cases, the sequence TPSIYFL should be very low because it only appeared in the negative screens; SSDAALH should be low because it was low in the reference library and high across all other screens; RVSTPPQ should be in the middle because it was high in a positive screen but also high in a negative screen; and GVTHKLQ should be very high because it was high in both positive screens and absent elsewhere; and so on for the remainder of the test cases. The predicted success of each sequence was compared to what occurred when the libraries were sorted with our software (actual ranking) and found to have good agreement (Fig 3B). Therefore, this test case validation illustrated the ability of our software to process data and order it according to ideal characteristics based on frequencies in a reference library, positive, and negative screens.

### Selectivity analysis identified sequences in a cellular context that were not found with available specificity software

After screening, specificity analysis, whereby a consensus sequence or motifs within the dataset are identified, can be an effective means of finding targeted sequences. Several types of software have been built around this principle, using statistics and alignment analysis to discover overrepresented motifs. This type of analysis is particularly effective when used with protein screens relative to in vitro cell screens because specific binding will correspond to fewer epitopes, leading to a greater overrepresentation of each motif within the data. We compared multiple published software programs by testing them on the data gathered from round two of our first streptavidin screen. In all cases, we were unable to directly input our barcode-segregated fastq files and instead had to write code to make a file that each program would accept. We were sometimes able to find the motif HPQ among other motifs (some of which were known to us as amplification biased sequences); for example, the software from the Heinis group found HPQ as the third identified motif (S1 Fig), while MEME and SLiMfinder software were unable to identify HPQ as a motif (S2 and S3 Figs). However, when we implemented the same software for round two of our first CAF screen, the results did not identify clear motifs outside of sequences we have found to have an amplification bias, presumably because any existing motifs are not as overrepresented due to a much greater availability of targets on cells than on a homogenous protein sample (S1–S3 Figs). Using the Heinis software, which bases motif-finding on both abundance and sequence homology, streptavidin screens resulted in much stronger motif identification than CAF screens (S1 Fig). As previously observed by the Heinis group [29], standard algorithms such as in MEME software tended to get stuck on high amplification clones, which could be due to the presence of sequencing errors that masquerade as similar sequences and make false consensus groups (S2 Fig); MEME was also limited to using 1,000 sequences and required an addition of a serine to each read in order to reach the minimum requirement of eight amino acids. Taken together, it is evident that the motif/consensus software elegantly identifies motifs from protein screens; however, these programs did not find suitable motifs in the cell screen data.

**Fig 4:**
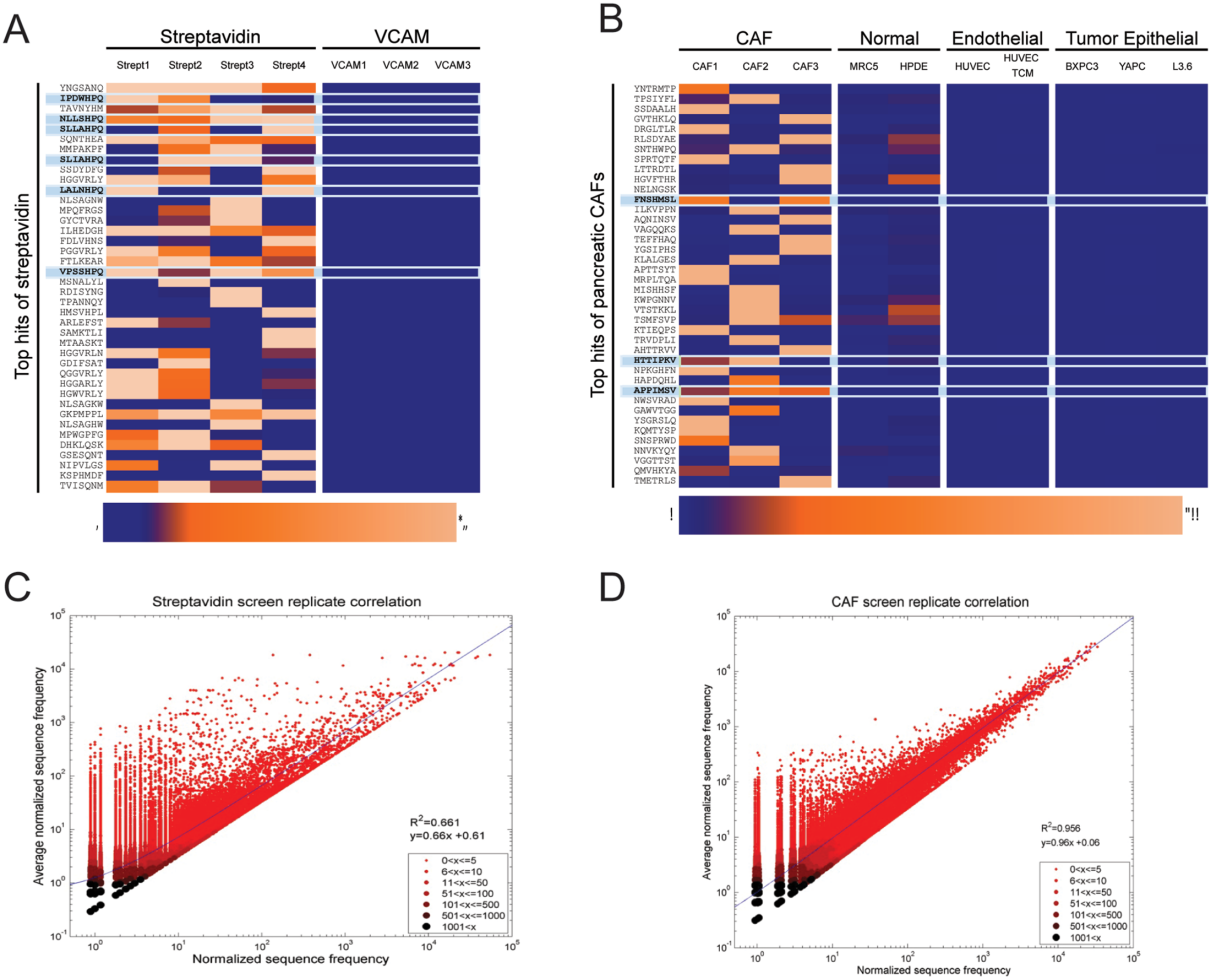
Comparison matrices. A heat map matrix visualization was generated using conditional formatting in Excel for the top 40 sequences from streptavidin (A) and CAF (B) sets of screens. The first two streptavidin screens used glycine to elute; whereas, the third and forth streptavidin screens were eluted with biotin. Scatter plots compare the frequencies and average frequencies of peptide sequences across independent screen replicates for streptavidin (C) and CAF (D) screens.

Additionally, most programs input one screen or programs compared a selected and unselected screen. Our software is able to fulfil a previously unmet need via selectivity analysis—comparing binding across many independent positive and negative screens. After using our software to process data from four streptavidin screens and three VCAM screens, six HPQ-motif sequences were listed in the top 20 sequences (Fig 4A). From our software analysis, we identified three potential CAF-selective sequences (FNSHMSL, HTTIPKV, APPIMSV) that were high in at least two of three positive screens and low in all negative screens (Fig 4B). The design of our sorting moves the selective sequences into the top fraction, but it is still necessary to visually inspect the top sequences to find sequences high in multiple positive screens and low in all others. Notably, none of the CAF sequences we identified nor motifs from these sequences were discovered in any other software analysis except our own. Indeed we found that our software is able to capture sequences enriched from the first to second round of biopanning even though our selectivity analysis is based entirely on round two data. In examining the change in the average normalized frequency in positive screens versus negative screens from the top 200 sequences identified by our software, we observed that these sequences tended to show a decrease in average normalized frequency for negative screens and an increase in average normalized frequency for positive screens (S4A and S4B Fig); however, we noted that not all sequences from the top 40 increased in normalized frequency from round one to round two (S4C and S4D Fig).

One contributing factor to the success of our analysis process is the ability to incorporate several positive screens, which is unique to our analysis approach. Using multiple positive screens accounts for the variability inherent in the phage display process that was evident when we plotted the frequency from each of three independent screens against the average frequency and found that there is much screen-to-screen variability inherent in the phage display process (Fig 4C and 4D). In contrast, variability from PCR amplification and DNA sequencing aspects of the process was much less, so the variability is likely stemming from the wet lab screening itself (S5 Fig). The degree of agreement between the three repeated screens as analysed by generating area overlap plots of the frequencies for sets of random sequences also supported the conclusion that robustness of the method was improved by basing candidate selection on multiple screens on the same protein, cells or tissue instead of a single screen (S6 Fig). Furthermore, our analysis technique is capable of eliminating sequences that bind as many negative targets as desired and can be easily re-sorted to use the same data to find targets of other cell types screened by categorizing a different set of screens as the positive screens (S7 Fig). Thus our software took into account the results of numerous positive and negative screens, including an unselected amplified reference library, to identify potential CAF-selective sequences (FNSHMSL, HTTIPKV, APPIMSV) that were not found with any other existing software.

### Two potential CAF-selective sequences (HTTIPKV and APPIMSV) validate in vitro and in vivo

**Fig 5:**
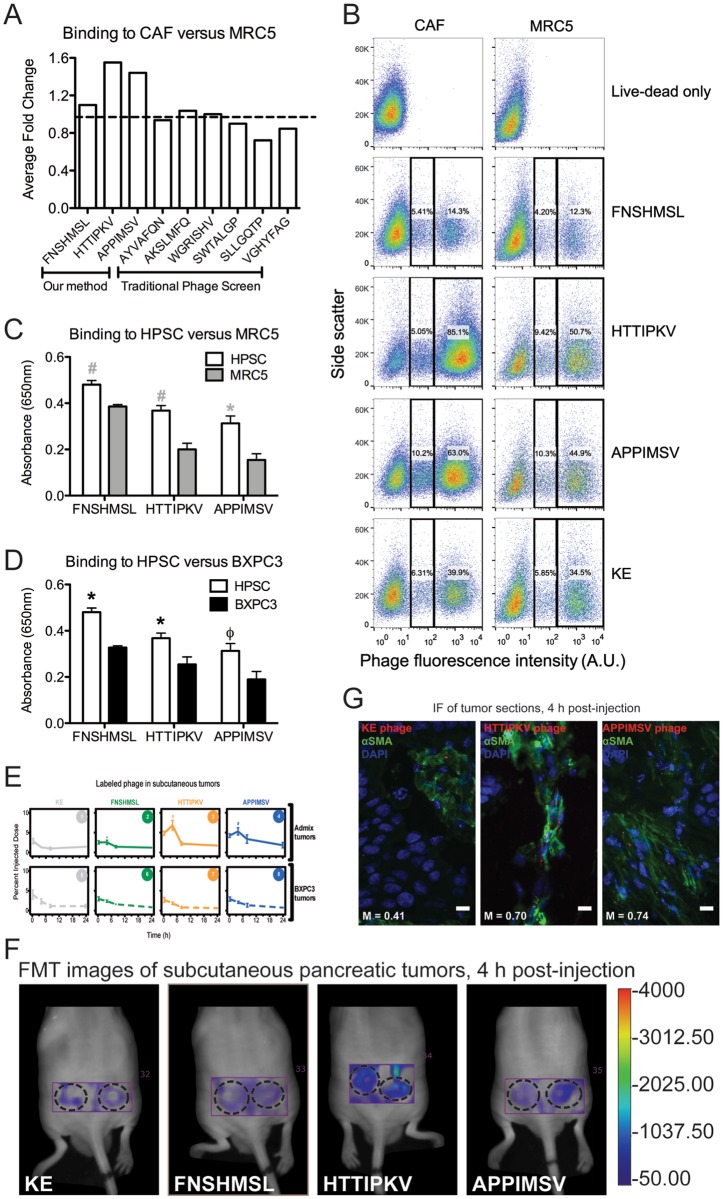
*In vitro* and *in vivo* peptide sequence validation. (A) An ELISA compares the binding of phage displaying the peptides to CAFs versus normal fibroblasts (MRC5). The first three sequences were selected using our selectivity analysis; whereas, the next six sequences were found using a traditional phage display approach. The dashed line indicates a fold change of 1.2. (B) Flow cytometry was performed by binding fluorescently-labeled phage to cells with a live-dead violet stain. Data was gated on cell population, live cells, and phage positive cells. (C) An ELISA compares binding of phage to HPSC and MRC5. Statistical significance was measured with a student t-test between HPSC and MRC5 where ^#^p<0.01 and *p<0.02. (D) An ELISA compares binding of phage to HPSC and BXPC3. Statistical significance was measured with a student t-test between HPSC and BXPC3 where *p<0.02 and ^Φ^p<0.06. (E) Fluorescently-labeled phage were injected into mice bearing subcutaneous admix CAF/BXPC3 tumors or BXPC3-only tumors (n = 6 tumors per group) and tumor accumulation was measured on an FMT using a region-of-interest around the tumor area. Statistical significance was determined using student’s t-test of each type of displayed peptide versus KE with ^#^p<0.01 and *p<0.02. (F) FMT images of mice with admix CAF/BXPC3 tumors scanned 4 h post-injection. Tumor regions have been circled with dashed lines. (G) Tumor sections of admix tumors were fixed, sectioned, and stained with anti-αSMA (green), then mounted with prolong gold anti-fade with DAPI (blue). The fluorescent labeling of the phage is colored red. Mander’s correlation coefficients (M) are indicated at the bottom of each image. For each phage type, images are representative of two tumors, three tumor sections each. Scale bars, 10 um.

ELISA experiments are a quick means of initially assessing if sequences are truly targeted or may have lingered in the screening process through non-specific interactions. ELISAs can be used with phage displaying peptides in order to reduce validation cost since sequences can be cheaply and quickly cloned into phage whereas peptide synthesis is more expensive and time-consuming. However, because the peptides are displayed in the context of a complete bacteriophage, they are subject to the high background binding exhibited in these assays by phage on cells. Historically, we have found that a 1.2 or higher increase in phage binding to target over non-target indicates that that phage should be further studied to assess targeting whereas all phage under that mark can be eliminated as non-specific binders. In an ELISA experiment, two out of three potential CAF-targeted sequences selected using our analysis approach showed at least 1.5-fold greater selectivity for CAFs than normal fibroblasts (MRC5) (Fig 5A). By comparison, zero out of six clones from a traditional four round phage display screen met the 1.2-fold standard (in traditional phage display, Sanger sequencing is used to identify clones picked by hand after the final round of screening so the final result is simply a group of phage that potentially bind the target with no associated quantitative data).

We further assessed the targeting of our sequences using flow cytometry and found that the two sequences (HTTIPKV and APPIMSV) continued to show higher binding to CAFs over normal fibroblasts (MRC5) (Fig 5B). We used wild-type KE phage (no displayed peptide) to determine background from the phage itself for each cell type assessed. The KE binding suggests that it was our selected peptides that increased the binding of the phage to the CAFs, while the small increase in binding to normal fibroblasts was likely attributable to nonspecific phage binding (Fig 5B). An additional ELISA using the cell line human pancreatic stellate cells (HPSCs) [43], of which the majority are CAFs, demonstrated that the potential CAF-targeted sequences were also selective for HPSCs (Fig 5C). Since the HPSCs were isolated from a different patient population across the country from the CAFs used in the biopanning, we concluded that the targets we were binding to were potentially expressed in cancer specimens and not limited to one patient. We also confirmed that our potential CAF-targeted sequences were not selective for tumor epithelial cells (BXPC3) (Fig 5D).

Next we evaluated the *in vivo* targeting of our potential CAF-targeted sequences with fluorescence molecular tomography (FMT) following systemic injection of the fluorescently-labeled phage into athymic nu/nu mice bearing subcutaneous BXPC3/CAF admix tumors or BXPC3-only tumors on their flanks. Admixing BXPC3 with CAF cells in a subcutaneous model produced tumors with a higher stromal content than injection of BXPC3 cells alone (S8 Fig). All three chosen sequences exhibited a classic pharmacokinetic curve on admix tumors; whereas, wild type showed no accumulation phase (Fig 5E, graphs 1–4). In BXPC3-only tumors, a clearance phase was observed for all phage and they overlapped with M13KE (Fig 5E, graphs 5–8). Phage accumulation was highest at 4 h where HTTIPKV and APPIMSV sequences had 3- and 2-fold higher binding, respectively (Fig 5E and 5F).

Immunofluorescent images of tumor sections from admix tumor mice sacrificed at 4 h show binding of HTTIPKV and APPIMSV phage to the stroma as assessed by one CAF marker, α-smooth muscle actin (αSMA), with minimal binding present for wild type KE phage (Fig 5G). Using the JACoP plugin (http://rsb.info.nih.gov/ij/plugins/track/jacop.html, [44]) in ImageJ, an average Mander’s correlation coefficient was calculated for 10 field views of three tumor sections of two tumors (60 images total) between the phage and αSMA-positive areas (representative images, Fig 5G). The Mander’s coefficient was high for both HTTIPKV (0.70) and APPIMSV (0.74) and low for KE (0.41) indicating phage clone binding to αSMA-positive cells. Taken together, these data indicate the binding of the HTTIPKV and APPIMSV to the CAF cells in vivo.

**Fig 6:**
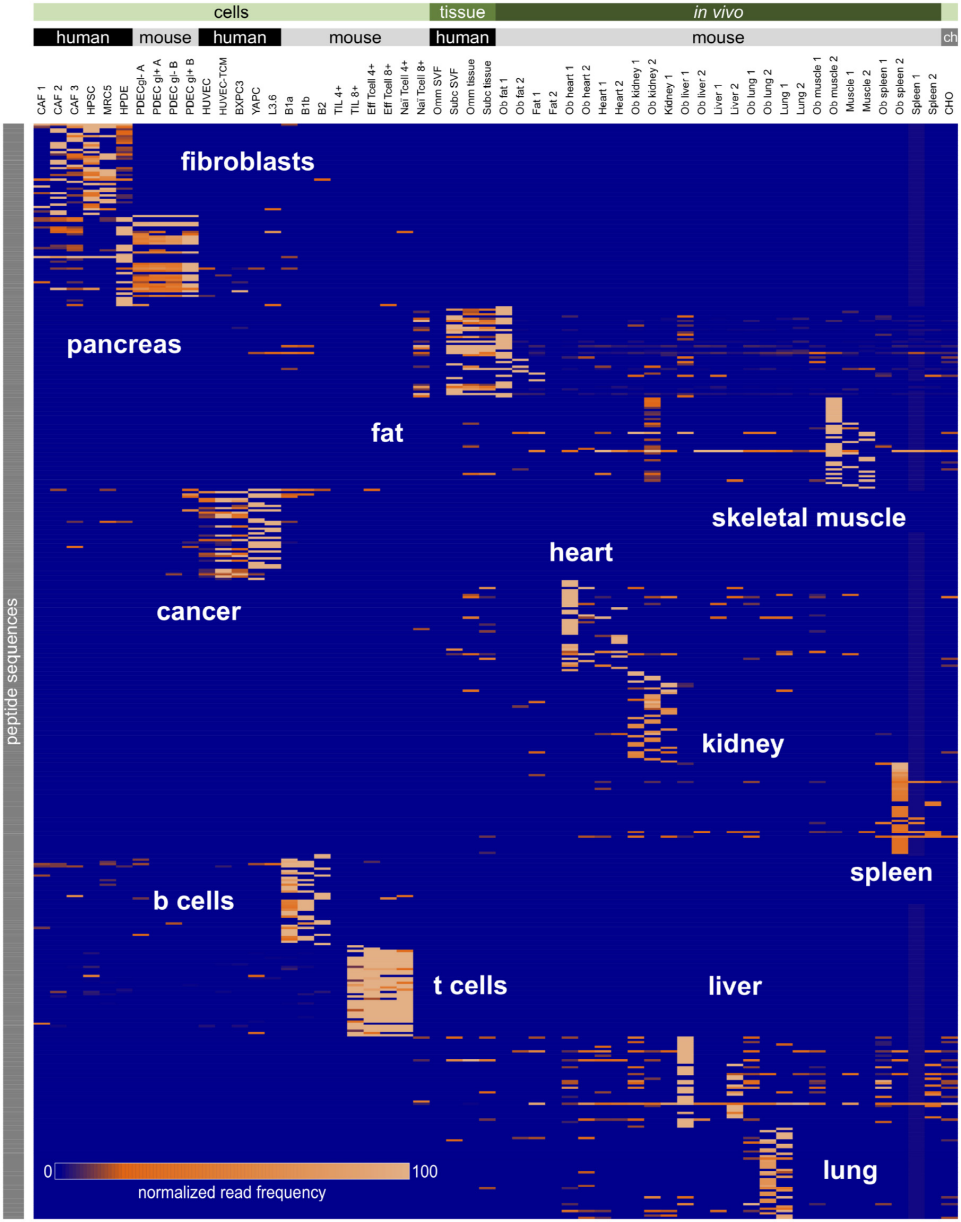
Peptide signatures of various cells and tissues. Peptides identified from screens performed on cell lines, ex vivo tissue specimens and in vivo screens were processed and analyzed using PHASTpep. They are presented as a heat map generated via conditional formatting in Excel. PDEC, pancreatic ductal epithelial cell; gl, glucose; B, b cells; TIL, tumor infiltrating lymphocyte; Eff, effector; Omm, ommental; SVF, stromal vascular fraction; Ob, obese; CHO, chinese hamster ovary.

As a final validation of selectivity, we ensured that the selected peptides (HTTIPKV and APPIMSV) do not bind any of the other cell lines or tissues that we have screened thus far. Using commercially available phage display libraries, we performed *in vitro* or *in vivo* selection followed by our selectivity analysis to identify peptides selective for each cell or tissue type (Fig 6). In total, we have screened 56 cells or tissues by performing two rounds of biopanning with a PhD 7 amino acid phage library on indicated cells or *in vivo* conditions and deep sequencing the DNA isolated post-screening. We compiled the resultant NGS data (84,232,601 reads) into a quantitative matrix that can be used as a reference to pre-emptively find other cells or tissues to which sequences may bind when used in vivo. Using this large dataset, we were able to search the sequences HTTIPKV and APPIMSV for other potential binding sites and found that the sequences did not have high normalized frequencies in any of these cells or tissues. Thus, we expect that our CAF-targeted sequences, HTTIPKV and APPIMSV, will bind exclusively to the CAFs and could be used systemically for targeted drug delivery vehicles or imaging agents.

## Discussion

Deep sequencing techniques have led to a fulsome characterization of the diversity and biases present in phage display libraries and heightened the potential of phage-display combinatorial libraries by addressing many of the weaknesses of the traditional technique. The advent of these large sets of sequencing data has led to many approaches to finding targeted motifs and consensus sequences. Yet, for cell or tissue screening and applications related to targeted drug delivery or imaging where the peptide will be eventually used systemically, researchers are interested not only in finding a peptide that targets a specific cell or tissue type, but also in finding a peptide that does not bind sites outside of their target-of-interest. Herein, we add to the repertoire of available analysis tools by presenting an approach and analysis software for finding highly selective sequences.

Our system starts with biopanning of a target-of-interest, for example a certain cell type, which is considered the positive screen. Multiple independent positive screens are carried out in order to account for the inherent variability in the phage screening process and increase the rate of successful candidate peptide identification. Biopanning of all negative targets, such as other cell types physiologically relevant to the positive target cell type, is also completed. Additionally, we amplify and sequence an aliquot of the unselected (naïve) library to serve as a reference. We originally anticipated using already available translation software for the initial processing of our data, but found that the differences between the fastq files produced by the Illumina sequencer (already separated by barcode) and those of other deep sequencing systems (not yet separated by barcode) were not immediately compatible. Thus it became more expedient to facilitate processing and translation of the type of data files we had by writing our own code to take care of these steps and this was incorporated as part one of our software. We also sought to provide as much flexibility as possible in our software so that users could tailor it to their individual needs. Therefore, we made it possible to simply select that NEB libraries were used or to enter custom library settings, use peptides of any size, and include up to twenty screens using the GUI provided (or by mimicking our provided script, to include as many screens as desired).

The second part of our software was tailored to our particular application of phage display: finding peptides that bind diseased cells over healthy cells for molecular imaging and targeted drug delivery. Phage display has demonstrated utility in a wide variety of other applications, including epitope mapping, protein-protein interaction analysis, enzyme inhibitor discovery, finding novel receptor agonist or antagonists, and vaccine development [45]. As a reflection of the variety of applications, there are diverse goals associated with software that has been developed for processing phage display deep sequencing data. Many tools use alignment analysis and statistics to identify motifs or find a consensus sequence within the data [29, 16–19, 35–36]. Other tools are used to find enrichment ratios for selected versus nonselected libraries [36–37], assess affinity when using FACS sorting [38], or reduce biases using continuous flow magnetic sorting combined with statistical analysis [21]. But for the purpose of targeting, we sought to create in silico mimicry of the exposure of the targeted peptide when it is administered systemically. This led us to develop a system in which the researcher could, in a premeditated fashion, designate any cell types that were important to avoid. Thus, our approach is designed to facilitate comparison of results across many different screens. Our software carries out a two-step normalization to total read number and the reference library and then sorts the data by the average ratio of positive to negative normalized read frequency. The result is a list of peptide sequences that are high in the positive screens and low in negative screens. With the screen-to-screen variability in mind, we elected to first validate sequences high in at least two out of three positive screens and low in all negative screens. We validated the targeting ability of two out of three selected sequences (HTTIPKV and APPIMSV) both on cells and in mice. Since our methodology finds targets to any cell type of interest in a short timeframe, it is also potentially applicable in personalized medicine to generate patient-tailored drug delivery systems from patient derived samples.

Because so many sequences are eliminated for being high in negative screens, in addition to removing negative target binders, this analysis also naturally discounted many false positives (nonspecific binders) as they also bound nonspecifically to a negative target. Seeing a more holistic view of screening data with many screens laid out side-by-side also led us to the discovery of “ubiquitous” binders, which are distinct from parasitic sequences [9] in that they are not necessarily gaining an advantage by faster amplification, but instead have their target expressed on all cell types or seem to be prone to nonspecific interactions. An additional re-sorting revealed ubiquitous binders that appear with increased frequency in all screens, most notably sequences closely related to YGAKDNL (S7 Fig). In the circumstance that negative screen data was not available, these sequences would have appeared as false positives that seemed to have good binding to the target. We have found that our selectivity analysis enhances the predictive power of phage display in two ways: (1) by requiring the sequences to be very selective (bind one cell type over many similar cell types), many nonspecific binders are removed, and (2) from the outset, the system pre-emptively searches for the highly selective peptides that will be useful in targeting applications.

Several groups have proposed using only one round of sequencing in order to avoid the biases that arise from bacterial amplification [21, 28–29]. This can be an effective means of reducing false positives. However, we have found that the success of using of only one round of biopanning is dependent on the target-of-interest. With protein targets, and even some cell targets, one round of biopanning seemed to be sufficient. Yet, we did still see significant enrichment of the HPQ-motif within our streptavidin screen from an additional round of biopanning. We found that screens performed on other cells types, including CAFs, or on ex vivo tissue samples required an additional round of biopanning because there was a higher degree of non-specific binding. We further found that there is valuable information contained in the comparison of frequency data from one round to the next that may be useful in selecting candidate peptides. Four out of the top ten sequences in our streptavidin screen contained the HPQ motif. When looking at the round two to round one ratio of the sequences, the HPQ-motif sequences had fold changes ranging from 35 to 65, whereas those of the other sequences ranged from 0.4 to 1.5 (S4C Fig). In comparing sets of round one and round two data, we became convinced that it informative to include two rounds of biopanning.

Through the process of running our first sets of screens, we noted that when ordered by read frequency, many of the top hits are the same for very diverse screens. This aligned well with the amplification bias and presence of parasitic sequences that have been beautifully described by the Derda group [9]. As we performed additional screens, we noticed that use of a new vial of PhD7 library seemed to have altered the top hits, possibly following the Derda group’s hypothesis that there is lot-by-lot variation in phage display libraries [9]. We additionally discovered that when using the Illumina sequencer, sequencing errors occur, causing peptides to be misappropriated to the incorrect screen at a lower frequency. Also, sequences with high frequency are found at a low frequency in all other libraries within a single multiplexed run. Thus it became our standard practice to always include a new reference standard (unselected, amplified library) with each run.

In this work, we have devised a strategy and provided software for finding highly selective peptide sequences using phage display and deep sequencing. Our process decreases the rate of false positives and rapidly highlights ideal candidate sequences. Using our approach, we found peptides targeted to the CAFs that could not be identified with other available analysis software or using motif finding logic. Our methodology is particularly applicable in the field of targeted imaging and drug delivery because the in silico comparisons allow elimination of non-selective peptides prior to validation experiments.

## Materials and Methods

Supplemental methods can be found in the S1 Appendix.

### Cell lines

CAFs (gift from Diane Simeone), YAPCs (ATCC, Manassas, VA), and BXPC3s (ATCC) were grown in RPMI medium 1640 (Life Technologies, Carlsbad, CA). MRC5s (ATCC) were grown in MEM medium (Life Technologies). HPSCs (gift from Rosa Hwang) and L3.6pls (ATCC) were grown in DMEM (Life Technologies). The RPMI, DMEM, and MEM media were supplemented with fetal bovine serum (10%), L-glutamine (2 mM), penicillin (100 units/mL), and streptomycin (100 ug/mL). HPDEs (gift from Craig Logsdon, MD Anderson) were grown in keratinocyte serum-free media with accompanying supplements (Life Technologies). HUVECs (LONZA, Basel, Switzerland) were grown in EBM supplemented with an EGM bullet kit (LONZA). Tumor-conditioned media (TCM) was harvested from L3.6pl cells following 48h of growth to ~90% confluence, and concentrated with Amicon 10k centrifuge filter. Concentrated media was added to the TCM HUVECs at a final concentration of 1x, 36 h prior to phage screening. For biopanning, cells were grown in a row of a 96-well plate in 1:2 serial dilutions and allowed to grow in a humidified, 37°C, 5% CO_2_ incubator. Plates were then examined under a microscope and wells with 70% confluence were used for biopanning. For ELISA experiments, 50,000 cells were plated in each well of two rows of a 96-well-plate and allowed to grow in a humidified, 5% CO_2_ incubator overnight.

### Biopanning

Protein was plated according to manufacturer’s instructions (NEB, Ipswich, MA). Cells were prepared as described above. Media was aspirated from each well and cells were rinsed with DPBS (HyClone, Logan, UT; with calcium and magnesium). 100 uL of DBPS/1% bovine serum albumin (BSA) containing 10 uL of PhD7 library (2e11 phage) was added to each well. Plates were returned to the humidified, 5% CO_2_ incubator. After 1 h, plates were removed and the supernatant was aspirated from the wells. Cells were washed five times with DPBS/1% BSA in order to remove non-specifically bound phage. The remaining phage were eluted for 9 min with 100 uL glycine buffer (0.2 M glycine, 0.5 M NaCl, HCl to pH 2.2). The phage-containing elution was immediately neutralized with 17 uL of 1 M Tris-HCl (pH 9.2).

### Naïve reference library preparation

10 uL of a 1:1000 dilution of PhD7 library (NEB) was used to create a reference library. Note: this aliquot must come from the same lot number of library due to the possible variability present between lot numbers [9]. This aliquot was amplified and prepared for deep sequencing in the same manner as all other libraries obtained by biopanning.

### PCR amplification of phage DNA

Phage were amplified and DNA isolated according to manufacturer’s protocols (NEB). A master mix was kept on ice (per sample): 2 uL universal primer (10 uM, see Table 1), 25 uL MyTaq HS Red Mix (2x, Bioline, London, UK), 19 uL MilliQ water. 46 uL of master mix was aliquoted into PCR tubes, to which the following was added: 2 uL indexing primer (10 uM, see Table 1) and 2 uL DNA template (1 ng/uL). All primers were designed to work with PhD7 or PhD12 libraries (NEB). Following an initial start of 95°C for one min, 20 cycles of PCR were carried out as follows: 95°C for 30 s, 60°C for 30 s, 72°C for 30 s. The PCR product was kept at 4°C until ready for purification. PCR purification was carried out using a QIAquick PCR purification kit (Qiagen, Hilden, Germany), following the manufacturer’s instructions. Samples were then prepared and sent to a core facility for Sanger sequencing to confirm proper indexing of the samples using PCRPrimerN FWD (10 uM, see S1 Table).

### PHASTpep of deep sequencing results

Samples were sent to the UVA Biomolecular Research Core Facility for deep sequencing. At the core facility, the quality and quantity of individual amplicons was assessed using Qubit fluorescence spectrophotometry in combination with an Agilent Bioanalyzer. Samples were run on an Illumina MiSeq Sequencer using the standard protocols recommended by Illumina for MSeq, using the MiSeq 150 V3 kits. Files obtained from deep sequencing (fastq) were entered into part 1 of the PHASTpep program with NEB PhD library selected and the number of amino acids per sequences entered as 7. This produced an excel file for each screen with sequences and corresponding read frequencies. Each excel file was then entered into part 2 of PHASTpep to obtain a matrix of sorted, normalized read frequencies and corresponding sequences. A heat map was generated using conditional formatting in Excel in order to visualize this matrix. Software, example files, and a README file are available on GitHub: https://github.com/LindseyBrinton/PHASTpep.git. Raw data files of deep sequencing are available in the NIH Short Read Archive: PRJNA316731 (SAMN04589895-SAMN04589912).

### Cloning into phage vector

DNA sequences corresponding to peptides were cloned into the M13KE vector (NEB). Oligos corresponding to the following sequences were chemically synthesized (Euorfins, Luxembourg) and ligated: (coding) [PHOS]GTACCTTTCTATTCTCACTCT(XXX)_7_GGTGGAGGTTC, and (non-coding) [PHOS]GGCCGAACCTCCACC(XXX)_7_AGAGTGAGAATAGAAAG, where (XXX)_7_ represents the DNA sequence of the desired peptide. DNA sequences of peptides were determined using codon optimization for k12 *E*. *coli*.

10 uL of 100 uM coding and non-coding solutions were combined with 30 uL of 17 mM MgCl_2_ in Qiagen EB, heated for 10 min in a 95°C water bath, and slowly cooled to room temperature. The M13KE vector (NEB) was digested with ACC651 and EagI-HF (NEB) followed by CIP. Vector was purified using the Qiaquick kit (Qiagen) according to manufacturer’s instructions with a final elution into 40 uL of EB. T4DNA ligase, M13KE vector, annealed oligonucleotides, and MilliQ water were combined so that there was a 6:1 ratio of oligonucleotide to vector. One reaction mix was prepared without insert as a ligation control. The reactions were ligated at room temperature for 15 min and heat inactivated via incubation at 70°C for 10 min. Transformed cells were plated on LB-tet plates for each reaction and the positive transformation control (made with pUC19 DNA) was plated on an LB-ampicillin plate (100 ug / mL ampicillin). After overnight incubation (37°C), colonies were picked and put in 50 uL MilliQ water. Samples underwent PCR (25 uL HS Taq red mix, 1 uL forward primer (5’-CCTTTAGTGGTACCTTTCTAT-3’, Eurofins), 1 uL reverse primer (5’-GCCCTCATAGTTAGCGTAACG-3’, Eurofins), 18 uL MilliQ water, 5 uL DNA from plaques in water) with 1 cycle of 95°C for 15 min, followed by 40 cycles of [95°C for 1 min, 55°C for 1 min, 72°C for 1 min], then 1 cycle of 72°C for 10 min. Sanger sequencing (Eurofins) was used to confirm insertion of the correct peptide. Clones with the correct sequence were amplified as described above and used in phage validation experiments.

### *In vitro* phage-based validation

1-step ultra tetramethylbenzidine (TMB)-ELISA substrate solution (ThermoFisher Scientific, Middletown, VA) was allowed to warm to room temperature. Cells were prepared as described in cell preparation. Cells were washed with DPBS+/1%BSA, 100 uL per well. For each type of phage and control wild-type KE phage, three wells were incubated with 40 uL of the phage (10e8 phages/uL). Phage were incubated on cells for 1 h in a humidified, 37°C, 5% CO_2_ incubator. Cells were then washed three times (DPBS+/1%BSA, 100 uL) and fixed (100 uL of 2% PFA, 5 min), then washed twice more. HRP-αM13 pIII monoclonal antibody (100 uL, 1:3000 dilution in DPBS+/1% BSA, NEB) was added to each well for 1 h. Cells were washed four more times and 100 uL of TMB was added. After the TMB reacted with the HRP, the absorption was measured on a microplate reader at 650 nm. Flow cytometry was performed by binding fluorescently labeled phage (Vivotag 645, PerkinElmer, Waltham, MA [31]) to CAF or MRC5 for 30 min. Cells were washed three times and live-dead violet stain (Life Technologies) was added. Data was gathered on Beckman Coulter CyAN ADP LX and gated on cell population, live cells, and phage positive cells using FlowJo software.

### In vivo phage-based validation

#### Mouse tumors

6–8 week old athymic nu/nu mice (Harlan, Indianapolis, Indiana) were anesthetized with isoflurane and injected with a total volume of 100 uL/tumor containing 2 million cells. For BXPC3-only tumors, 2 million BXPC3 cells were combined 1:1 with Matrigel (BD Biosciences, Franklin Lakes, NJ). For admix tumors, 0.5 million BXPC3 cells and 1.5 million CAF cells were combined 1:1 with Matrigel. Two tumors were subcutaneously injected per mouse, on each flank, and allowed to grow for 2.5 weeks before the start of the study. Tumor size and mouse weight were measured twice weekly. Mice with tumor burden in excess of 1000 mm^3^ or displaying clinical signs of severe illness, including weight loss >10%, inability to get food, extreme lethargy, or failure to return to normal activity, would be euthanized using compressed carbon dioxide gas, followed by cervical dislocation. Eight groups total (n = 6 tumors per group) were used for this study: admix KE, admix FNSHMSL, admix HTTIPKV, admix APPIMSV, BXPC3 KE, BXPC3 FNSHMSL, BXPC3 HTTIPKV, and BXPC3 APPIMSV.

#### Quantitative fluorescence imaging

Phage with targeted displayed peptides and control wild-type KE phage were labeled as previously described [1]. Briefly, fluorescent dye (Vivotag 680, PerkinElmer) and amplified phage were incubated and free dye removed using PEG precipitation and microfugation (14,000 rpm, 10 min). The phage were suspended in 1 mL of DPBS, PEG-precipitated again, and suspended in 200 uL DPBS. Mice received tail vein injections equal quantities of dye-labeled phage. At various time-points, isoflurane to effect was given as an inductional and maintenance anesthetic and mice were imaged in a fluorescence molecular tomography system (FMT, PerkinElmer). Images were reconstructed using the FMT software and regions of interest were drawn around the tumor areas to quantify the amount of dye (pmol) present in that area. All animal experiments were conducted under the supervision of the Office of Animal Welfare at UVA with UVA Animal Care and Use Committee (ACUC) protocol approval (protocol number 3731-10-14). Animal welfare was monitored by vivarium staff trained to identify and intervene for distressing health problems. The primary method of euthanasia was overdose of inhaled isoflurane and cervical dislocation was used to confirm death.

### Microscopy

Excised tumors were sliced in half to allow for immunoflurescence (IF) and immunohistochemistry (see Supplemental methods). To prepare tumor tissue for IF, the tumor was immersed in 4% paraformaldehyde for 20 min, submerged in optimal cutting temperature compound (OCT, Tissue-tek), and frozen with liquid nitrogen vapors. Frozen cassettes were immediately placed at -80°C for storage. Frozen tissues were cut using a cryostat, with sections 8um thick. OCT was removed from tumor sections frozen to glass microscope slides by submersing the slide in DPBS at room temperature for 5 min. Sections were then blocked for 30 min with 1% fish skin gelatin (FSG), rinsed twice with DPBS, stained with FITC-conjugated α smooth muscle actin (αSMA, 1:250, Sigma) for 40 min, and rinsed five times with DPBS. Microscopy was performed using an Olympus fluorescence microscope with a 60x oil immersion objective (1.25 NA) and QImaging Retiga 2000R camera with accompanying software.

## Supporting Information

S1 Appendix Supplemental methods. (DOCX)

S1 Fig Sequence logos generated by inputting our data into software from the Heinis group. MATLAB code was written to generate files compatible with the motif finding software available from the Heinis group [29] (https://github.com/LindseyBrinton/PHASTpep.git, in the “software adaptations” folder). (A) Running these files from the Streptavidin1 screen resulted in 21 logo groups. (B) Running these files from the CAF1 screen resulted in identification of 18 logo groups.
(JPG)

S2 Fig Sequence logos from MEME. To enable use of the MEME software [16], we used Excel to generate FASTA files following the guidelines of the software, using the top 1,000 sequences and adding a Serine to the end of each sequence since the minimum allowed sequence length is 8 amino acids. The software was run in normal mode with a site distribution of any number of sequences and was set to search for 10 motifs.
(JPG)

S3 Fig SLiMfinder motifs. Fasta files were created of the top 200 sequences from screens of streptavidin, CAF, and their associated reference libraries (https://github.com/LindseyBrinton/PHASTpep.git, in the “software adaptations” folder). SLiMfinder [18] was run using the reference library file for the amino acid distribution and with the following settings: efilter = F sigcut = 1.0 topranks = 10 combamb = T masking = F maxseq = 10000. The top ten motifs generated from streptavidin (A) and CAF (B) are listed.
(JPG)

S4 Fig Round 1 to round 2 library enrichment. A plot was generated using MATLAB showing the trajectory from round one (blue) to round two (red) of the top 200 sequences of the Streptavidin screens (A) and CAF screens (B). The y-axis is the average normalized read frequency of negative screens with higher frequencies indicating more binding to non-target. The x-axis is the average normalized read frequency of positive screens with higher frequencies indicating more binding to target. The ratio of average round two normalized frequencies to round one average normalized frequencies was calculated for the top 40 sequences of the Streptavidin screens (C) and the CAF screens (D).
(JPG)

S5 Fig PCR and DNA replicate correlation. Scatter plots compare the frequencies and average frequencies of peptide sequences across replicates at the PCR step (A) and DNA sequencing step (B).
(JPG)

S6 Fig Area overlap plots. In MATLAB, a random number generator was used to pull out 200 sequences from the three screen repetitions and the reference library for Streptavidin (A) and CAF (B). The data was visualized as area plots with lightly shaded regions that show overlap between different screens.
(JPG)

S7 Fig Re-sorting of matrix data. Re-sorting of data yields targeted sequences for VCAM (A), as well as pancreatic tumor epithelial cells, and identifies ubiquitous binding peptides (B). Re-sorting the matrix data using the VCAM or tumor epithelial screens as positive screens and the other screens as negative screens results in new sets of targeted peptides. Similarly, by looking for peptides high in all screens, we found peptides that bind all of the cell types examined.
(JPG)

S8 Fig Stromal content of subcutaneously injected tumors. Light microscope images of HE stained admix CAF/BXPC3 or BXPC3 only tumor sections show stromal and epithelial compartments. 10x scale bar (black), 50 um. 40x scale bar (black), 10 um.
(JPG)

S1 Table The primers used for sequence indexing prior to deep sequencing. Universal primer was used with one indexing primer for each library. PCRPrimerN (FWD) was used to prepare samples for Sanger sequencing.
(JPG)
