# Supplementary material for: PHASTpep: Analysis Software for Discovery of Cell-Selective Peptides via Phage Display and Next-Generation Sequencing
Source: PLoS One. 2016 May 17;11(5):e0155244. doi: 10.1371/journal.pone.0155244 (PMC4871350; doi:10.1371/journal.pone.0155244)
Supplement: S1 Appendix — (DOCX) [file pone.0155244.s001.docx]

**Supplemental Methods**

**Motif software**

To use the program created by the Heinis group, it was necessary to write a script that formatted our data to use with their program since their program is written to first input non-segregated data and the Illumina sequencer automatically separated our files by barcode. A MATLAB script was written that translated our data and generated a .txt file that mimicked their input format of peptide sequence-abundance-nucleotide sequence (<https://github.com/LindseyBrinton/PHASTpep.git>, in the “software adaptations” folder). The data was then imported into Excel, sorted by abundance, and resaved as a tab delimited file. The file was then run through Clustering.m [17] using the 200 most abundant sequences.

For the MEME software, we generated fasta files in Excel per the guidelines of the software, which require only the top 1,000 sequences and a minimum peptide length of eight amino acids. In order to have eight, we added a serine to the end of every sequence. The MEME software [23] was then run in normal mode, with a parameter to search for 10 motifs, and allowing any number of sequences for the site distribution.

In order to use the SLiMfinder software, we wrote a MATLAB script that made fasta files of the top 200 sequences (<https://github.com/LindseyBrinton/PHASTpep.git>, in the “software adaptations” folder). The file was run through SLiMfinder [25], with the reference library file as the amino acid distribution and with the following settings: efilter=F sigcut=1.0 topranks=10 combamb=T masking=F maxseq=10000.

**Analysis of round one data**

Using MATLAB, the normalized frequencies across CAF screens were averaged separately for round one and round two. Likewise, the normalized frequencies across the negative screens were averaged for round one and round two. The 200 most abundant sequences from the round one data were plotted (red dots) with the average of the CAF screens as the x-axis and the average of the negative screens as the y-axis. The round two data was plotted in the same way (blue dots) and a line drawn between peptides that were in the top 200 of both the round one and round two data.

Area plots were also generated in MATLAB. A random number generator was used to pull out 200 sequences from a dataset. Normalized frequencies from individual screens were plotted for each sequence, including the reference library. Color was used to distinguish different screens, with a light shading so that overlap between multiple screens could be visualized.

**Immunohistochemistry**

To prepare tumor tissue for IHC, tumors were immersed in 10% formalin for 20 minutes and then in 70% ethanol for storage. Fixed tissue was given to the UVA Cardiovascular Research Center histological service, where the tissues were paraffin-embedded, sectioned, and stained with HE. Microscopy was performed using a Zeiss light microscope with either a 10x (0.25 NA) or 40x (0.65 NA) objective and Zeiss AxioCam MRc camera with accompanying software.
