# Supplementary figures and images for: PHASTpep: Analysis Software for Discovery of Cell-Selective Peptides via Phage Display and Next-Generation Sequencing
Source: PLoS One. 2016 May 17;11(5):e0155244. doi: 10.1371/journal.pone.0155244 (PMC4871350; doi:10.1371/journal.pone.0155244)

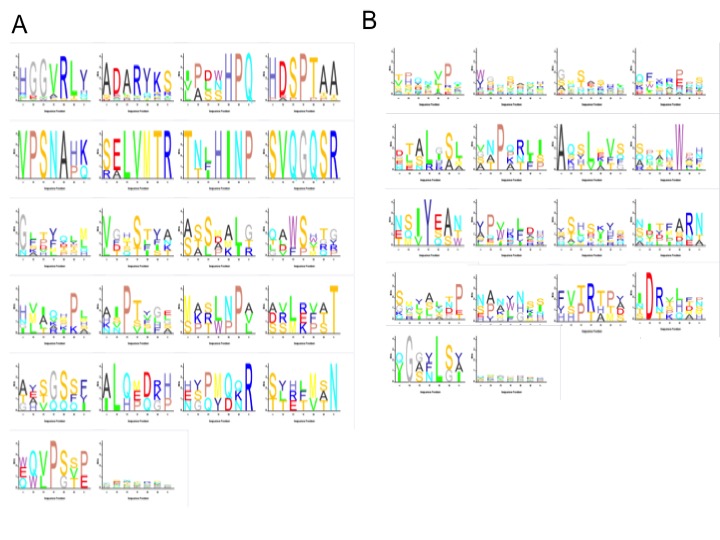

Supplement: S1 Fig — MATLAB code was written to generate files compatible with the motif finding software available from the Heinis group [29] (https://github.com/LindseyBrinton/PHASTpep.git, in the “software adaptations” folder). (A) Running these files from the Streptavidin1 screen resulted in 21 logo groups. (B) Running these files from the CAF1 screen resulted in identification of 18 logo groups. (JPG) [file pone.0155244.s002.jpg]

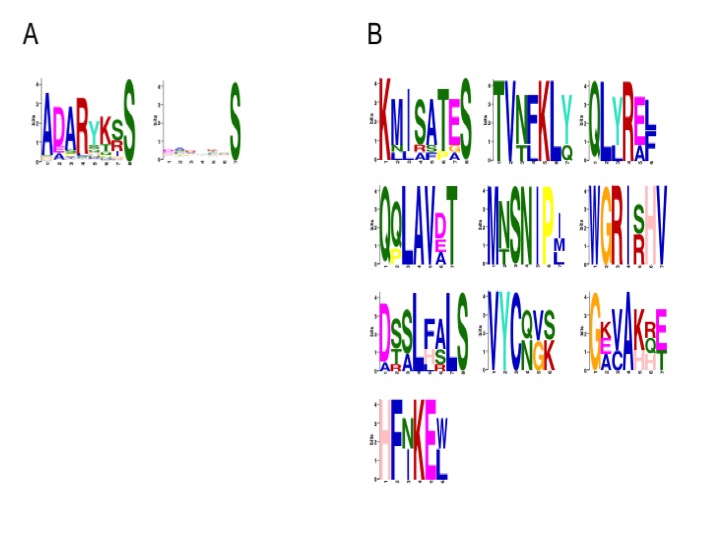

Supplement: S2 Fig — To enable use of the MEME software [16], we used Excel to generate FASTA files following the guidelines of the software, using the top 1,000 sequences and adding a Serine to the end of each sequence since the minimum allowed sequence length is 8 amino acids. The software was run in normal mode with a site distribution of any number of sequences and was set to search for 10 motifs. (JPG) [file pone.0155244.s003.jpg]

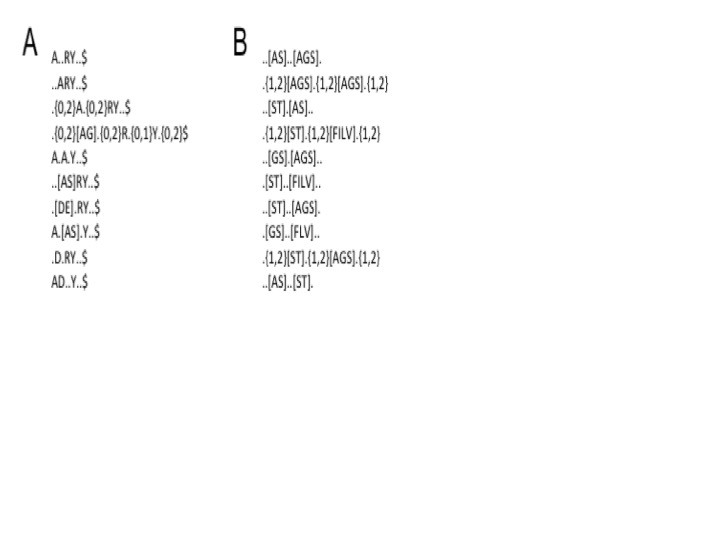

Supplement: S3 Fig — Fasta files were created of the top 200 sequences from screens of streptavidin, CAF, and their associated reference libraries (https://github.com/LindseyBrinton/PHASTpep.git, in the “software adaptations” folder). SLiMfinder [18] was run using the reference library file for the amino acid distribution and with the following settings: efilter = F sigcut = 1.0 topranks = 10 combamb = T masking = F maxseq = 10000. The top ten motifs generated from streptavidin (A) and CAF (B) are listed. (JPG) [file pone.0155244.s004.jpg]

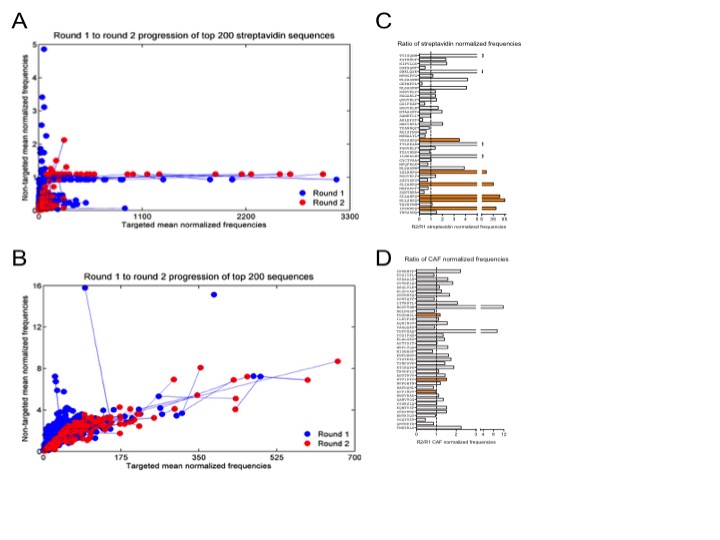

Supplement: S4 Fig — A plot was generated using MATLAB showing the trajectory from round one (blue) to round two (red) of the top 200 sequences of the Streptavidin screens (A) and CAF screens (B). The y-axis is the average normalized read frequency of negative screens with higher frequencies indicating more binding to non-target. The x-axis is the average normalized read frequency of positive screens with higher frequencies indicating more binding to target. The ratio of average round two normalized frequencies to round one average normalized frequencies was calculated for the top 40 sequences of the Streptavidin screens (C) and the CAF screens (D). (JPG) [file pone.0155244.s005.jpg]

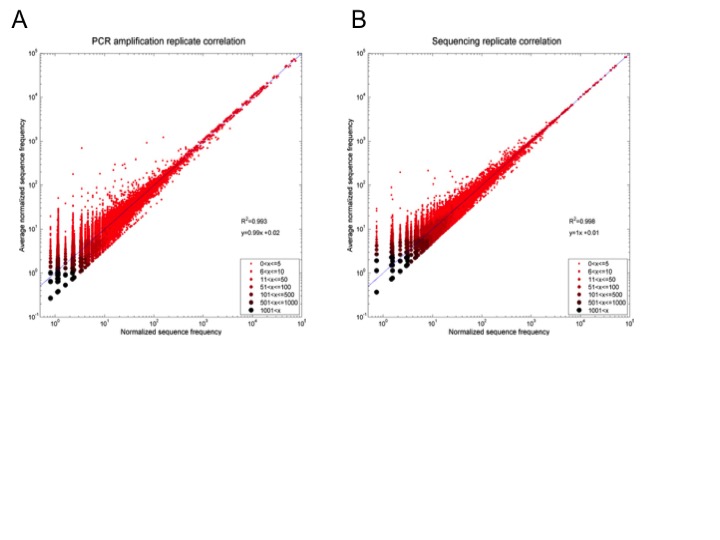

Supplement: S5 Fig — Scatter plots compare the frequencies and average frequencies of peptide sequences across replicates at the PCR step (A) and DNA sequencing step (B). (JPG) [file pone.0155244.s006.jpg]

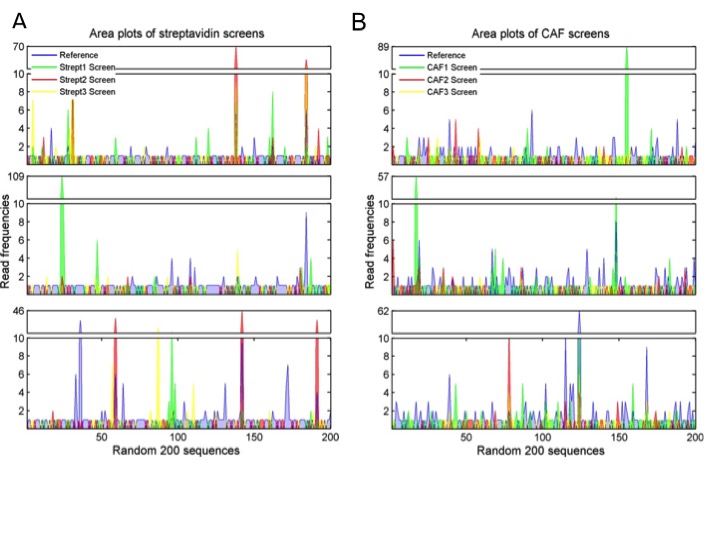

Supplement: S6 Fig — In MATLAB, a random number generator was used to pull out 200 sequences from the three screen repetitions and the reference library for Streptavidin (A) and CAF (B). The data was visualized as area plots with lightly shaded regions that show overlap between different screens. (JPG) [file pone.0155244.s007.jpg]

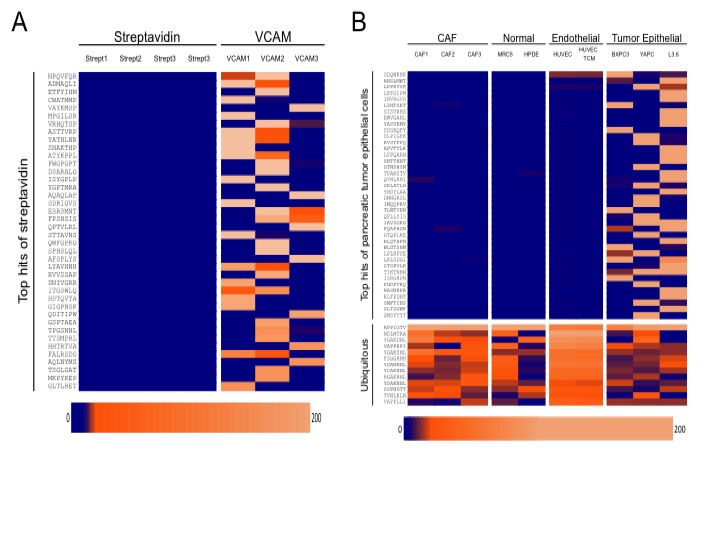

Supplement: S7 Fig — Re-sorting of data yields targeted sequences for VCAM (A), as well as pancreatic tumor epithelial cells, and identifies ubiquitous binding peptides (B). Re-sorting the matrix data using the VCAM or tumor epithelial screens as positive screens and the other screens as negative screens results in new sets of targeted peptides. Similarly, by looking for peptides high in all screens, we found peptides that bind all of the cell types examined. (JPG) [file pone.0155244.s008.jpg]

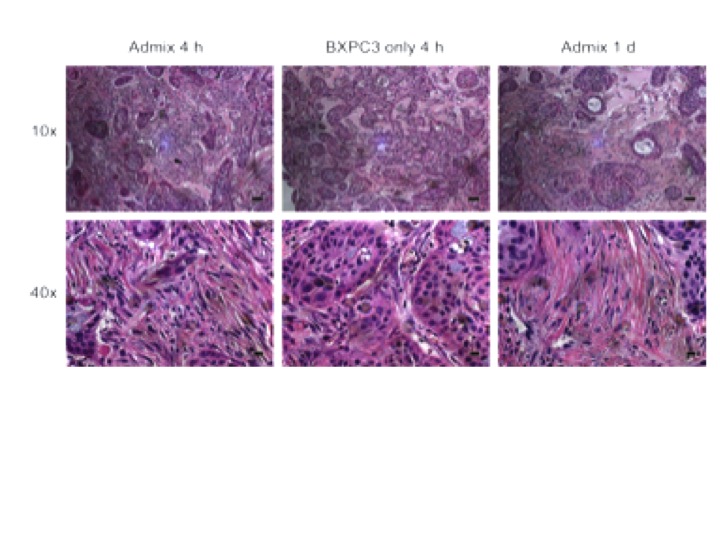

Supplement: S8 Fig — Light microscope images of HE stained admix CAF/BXPC3 or BXPC3 only tumor sections show stromal and epithelial compartments. 10x scale bar (black), 50 um. 40x scale bar (black), 10 um. (JPG) [file pone.0155244.s009.jpg]

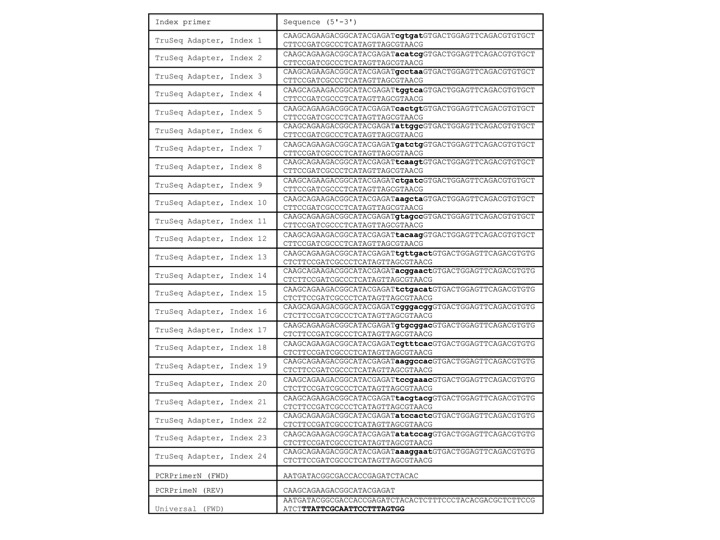

Supplement: S1 Table — Universal primer was used with one indexing primer for each library. PCRPrimerN (FWD) was used to prepare samples for Sanger sequencing. (JPG) [file pone.0155244.s010.jpg]
